# Wound of the Intestines, Successfully Treated

**Published:** 1850-03

**Authors:** Dandridge B. Hilliard

**Affiliations:** Halifax County, North Carolina


*Wound of the Intestines, successfully treated.* By DANDRIDGE B. HILLIARD, M. D., of Halifax county, North Carolina.

To the Editor of the Medical Examiner:

SIR,—I beg leave to send you the notes of a case of wounded intestine, with recovery. On the 11th May, at 9 o'clock, A. M., I was called to see a negro slave, belonging to Capt. Hardy Pitts, who had been stabbed in the left iliac region. On reaching him, I found that nearly all the small intestine had protruded, on a portion of which was a transverse cut, three-fourths of an inch in length, with an accompanying wound of the mesentery, one of the blood vessels of which was divided. The injury had been received about an hour before my arrival, in a conflict with another slave, who inflicted it with a sharp pointed knife, making an external wound two inches in length. I immediately administered to him 30 gtt. sul. ether, and then secured the bleeding vessel, which fortunately had been compressed by the weight of the protruded mass, by ligature. The wounded intestine, which displayed the mucous membrane everted, was closed with a fine suture needle, by neatly drawing together the outer edges without puncturing the mucous membrane, by this means approximating its edges, so that when the threads should be disengaged from the outer coat of the bowel by suppuration, the inner coat would be healed, thereby leaving no orifice through which matter could escape. The ends were cut off close, and the whole returned into the cavity of the abdomen. The external wound was then closed by stitches, sufficiently to retain the bowels, leaving a small orifice through which the blood might escape, which I favored by laying the patient in a favorable position, and preventing the bowels from plugging it up by the introduction of a finger. After which I ordered him thirty drops of laudanum, to be repeated every hour till composed. Three o'clock in the evening, complained of much nausea and restlessness; ordered him 20 drops sul. ether and 15 drops laudanum, after which he took some gruel, appeared more composed, and slept during the night, On the morning of the 12th, there was much restlessness; pulse 100; abdomen slightly tympanitic; ordered mucilaginous drinks; a cold corn meal poultice, with slippery elm, over which was poured an ounce of laudanum, and the whole of the abdomen covered over with it, with orders to renew it every fifteen minutes, and to give 30 drops sweet spts. nitre every hour till the fever abated. Two hours after, pulse 80, soft and compressible; ordered 35 drops laudanum. Slept tolerably well that night.

13th. In the morning expressed himself more comfortable; 4 o'clock in the evening complained of nausea; pulse 120; abdomen tympanitic and tender; much thirst; ordered 20 drops ether and 30 drops spts. nitre every half hour, to be followed by 35 drops laudanum; and an opium plaster around the umbilicus; the cold mush poultice applied as before, and renewed every fifteen minutes. In a few hours the pulse fell to 70, and the patient expressed himself comfortable. 14th. Some fever, which was soon subdued by the same prescription. On the 15th, 16th, and 17th, continued the same treatment, so far as the indications demanded it. On the 18th, ordered an injection of 12 oz. slippery elm tea, to be repeated in three hours. 19th. 12 oz. corn-meal gruel, 3 oz. castor oil, and 60 drops laudanum, by injection, which produced a copious evacuation, the first he had had since the accident. Three weeks after, the patient resumed his labors on the farm, and at this time (Dec. 3) is perfectly sound and healthy. Had I allowed the ends of the sutures to depend externally, where they would have been partially confined, the peristaltic motion of the bowel would have caused the stitches to tear out. My thanks are due to my highly valued friend, Dr. Thomas Arrington, of Nash county, for the efficient services rendered me in this case.

